# Molecular Cloning and Characterization of a New Family VI Esterase from an Activated Sludge Metagenome

**DOI:** 10.3390/microorganisms10122403

**Published:** 2022-12-04

**Authors:** Ren-Bao Liaw, Jo-Ching Chen, Mei-Ping Cheng

**Affiliations:** 1 Physiology Division, Livestock Research Institute, Council of Agriculture, Executive Yuan, Tainan 712009, Taiwan; 2 Breeding and Genetic Division, Livestock Research Institute, Council of Agriculture, Executive Yuan, Tainan 712009, Taiwan; 3 Deputy Director Office, Livestock Research Institute, Council of Agriculture, Executive Yuan, Tainan 712009, Taiwan

**Keywords:** metagenome, activated sludge, family VI esterases, regioselectivity

## Abstract

A new esterase gene, *est6*, was discovered in an activated sludge metagenomic library. The 729-bp gene encodes a 242-amino acid protein (designated Est6) with a molecular mass of 26.1 kDa. Est6 shared only a moderate identity to a putative hydrolase with the highest BLASTP analysis score. Most of the closely related proteins are uncharacterized and are predicted from genome sequencing data of microorganisms or metagenomic DNA sequences. The phylogenetic analysis of Est6 showed that the protein was assigned to family VI esterases/lipases. The catalytic triad of Est6 was predicted to be Ser135, Asp188, and His219, with Ser135 in a typically conserved pentapeptide (GFSQG) of family VI members, which was further confirmed by site-directed mutagenesis. The *est6* gene was overexpressed successfully in its soluble form in *Escherichia coli* and then purified to its tag-free form and homogeneity by affinity chromatography. The purified Est6 in pH 8.0 buffer was active as a monomer. The optimal conditions for Est6 activity were at a temperature of 45 °C and pH of 8.0 when using p-nitrophenyl acetate as a substrate. The enzyme was stable over wide temperature and pH ranges, and it exhibited activity in the presence of organic solvents, metal cations, or detergents. Furthermore, the enzyme showed significant regioselectivity in the spectrophotometric analysis. In conclusion, Est6 might have the potential for applications in biotechnological processes.

## 1. Introduction

Lipolytic enzymes are important biocatalysts for some industries, such as foods and feed, detergent, chemical synthesis, cosmetics and flavors, and pharmaceutical sciences [1,2]. Lipases (EC 3.1.1.3) and esterases (EC 3.1.1.1) are members of lipolytic enzymes that hydrolyze and synthesize long-chain and short-chain acylglycerols [3], respectively. Lipolytic enzymes possess special characteristics of broad substrate specificity, stability in organic solvents, and regio- and stereo-selectivity [2,4].

Bacterial lipolytic enzymes are classified into eight families (families I–VIII) according to a comparison of primary sequences [3]. For the advanced and cost-effective next-generation DNA sequencing technology, 19 families (family I–XIX) have been identified according to the deduced amino acid sequences of lipolytic enzymes [5]. The lipolytic enzymes of family VI, with a molecular mass of 23–26 kDa, are among the smallest esterases. The enzymes in this family have the conserved pentapeptide motif GFSQG throughout the sequence alignment. To date, only a few family VI esterases have been characterized in detail: esterase II from *Pseudomonas fluorescens* [6], a serine esterase from *Spirulina platensis* [7], intracellular esterase from *Pseudomonas aeruginosa* PAO1 [8], EstA6 from *Pseudomonas* sp. CR-611 [9], Esth from *Shewanella* sp. [10], and EstRag from *Lysinibacillus* sp. [11]. Almost all family VI lipolytic enzymes isolated from different bacteria have a high preference for short-chain substrates with broad specificity and demonstrate no activity towards long-chain triglycerides. However, very little is known about the characteristics of other conceptual lipolytic enzymes in this family.

Due to the development of metagenomic libraries from various complex samples such as soils [12,13,14,15,16], compost [17,18,19], activated sludge [20,21,22,23], and sediments [24,25,26], many novel lipolytic enzymes have been discovered. Tremendous genetic resources from a number of unculturable microorganisms might be utilized more effectively and successfully when the metagenomic approach is used in the discovery of novel genes.

In this study, we investigated a lipolytic clone (ASL06, accession number: FJ951170) previously isolated from an activated sludge metagenome [22]. A new esterase Est6 was encoded in this clone and determined to be a member of family VI lipolytic enzymes. Further study of the *est6* gene involved cloning it into an expression vector and over-expressing it as soluble proteins in *Escherichia coli*. After single-step purification with the Profinity eXact fusion-tag system, the tag-free enzyme Est6 was obtained. The purified Est6 was studied for its biochemical properties. A test of its positional specificity with a spectrophotometric method indicated that Est6 was regiospecific towards triacylglycerol.

## 2. Materials and Methods

### 2.1. Bacterial Strains and Plasmids 

*Escherichia coli* TOP10 (Invitrogen, Carlsbad, CA, USA) was used as a cloning host, and the expression hosts were *E. coli* BL21 Star (DE3) (Invitrogen, Carlsbad, CA, USA) and *E. coli* BL21(DE3) (Yeastern Biotech Co., Ltd., Taipei, Taiwan). The expression vector pPAL7 was purchased from Bio-Rad (Hercules, CA, USA).

### 2.2. General DNA Manipulations

Recombinant plasmid DNA was extracted using a commercial plasmid extraction kit (Wizard Plus SV Minipreps DNA Purification Systems, Promega, Madison, WI, USA). Restriction enzymes (Fermentas, Vilnius, Lithuania) and T4 DNA ligase (Roche Applied Science, Mannheim, Germany) were used according to the supplier’s instructions. General DNA manipulation techniques used in this study were applied as described by Sambrook and Russell (2001) [27].

### 2.3. Sequence and Phylogenetic Analysis

The lipolytic clone (ASL06, accession number: FJ951170) used in this study was previously isolated from an activated sludge metagenome [22]. Open reading frame (ORF) analysis for lipolytic clone ASL06 was performed using the ORF finder of NCBI (https://www.ncbi.nlm.nih.gov/orffinder/, accessed on 8 November 2022) [28]. The predicted function of Est6 was annotated using the BLASTP search against the NCBI non-redundant protein database [29] to search for the closest related protein and microorganism. The signal peptide was predicted using a SignalP 5.0 server (https://services.healthtech.dtu.dk/service.php? SignalP-5.0, accessed on 8 November 2022) [30]. The molecular mass and pI of Est6 were analyzed using Vector NTI Suite 11.0 package software (Invitrogen, Carlsbad, CA, USA). The solubility of Est6 was predicted by SOLpro analysis [31]. Multiple alignments of the amino acid sequences of the lipolytic enzymes and bacterial lipase/esterase reference families were analyzed using the Clustal X version 2.0 program [32]. The neighbor-joining phylogenetic tree of the lipolytic enzyme sequences was constructed with the molecular evolutionary genetics analysis package (MEGA, version 7.0) [33]. A bootstrap analysis with 1000 replicates was performed to check the robustness of the tree. Structural analyses of Est6 were carried out by PSIPRED 4.0 [34], I-TASSER [35], and ColabFold [36], respectively. ColabFold is a fast and easy-to-use software to accelerate the prediction of protein structures and complexes by combining the fast homology search of MMseqs2 with AlphaFold2 [37].

### 2.4. Cloning, Expression and Purification

A pair of primers including Est6F (5′-CCAAGCTTTG**ATG**ACTGAGCTGCGG ATCA-3′ and Est6R 5′-CCGGATCCCTAGTGCTCGGCCTCGGC-3′) with restriction cutting sites (sequences shown with underscores, start codon shown in bold) for *Hin*dIII and *Bam*HI, respectively, were used to amplify the full length of the putative esterase/lipase gene *est6*. The PCR mixtures contained a unique buffer mix J (FailSafe™ PCR PreMix Selection Kit, Epicentre Biotechnologies, Madison, WI, USA) for amplification of high-GC templates and a high-fidelity *Pfu* polymerase (*PfuUltra* II Fusion HS DNA polymerase, Stratagene, La Jolla, CA, USA). The PCR was programmed as follows: pre-denaturation for 5 min at 94 °C, the initial seven cycles of amplification at 94 °C for 45 s, 58 °C for 45 s, and 72 °C for 60 s; followed by 28 cycles of amplification at 94 °C for 45 s, 65 °C for 45 s, 72 °C for 60 s; and post-extension for 10 min at 72 °C using a thermal cycler (MyCycler, Bio-Rad, Hercules, CA, USA). The amplified DNA fragment was purified and digested with *Hin*dIII and *Bam*HI simultaneously. After purification, the amplified fragment was ligated to the expression vector pPAL7, treated with the same restriction enzymes, and transformed to *E. coli* TOP10. The recombinant plasmid was sequenced to confirm that the DNA sequence of the insert was identical to the original clone, with the proper frame of expression vector. The plasmid was designated as pPAL7-Est6. 

The recombinant plasmid pPAL7-Est6 was transformed into the *E. coli* BL21 Star (DE3) by heat shock (42 °C for 30 s) for overexpression. The recombinant *E. coli* was cultured in an LB broth containing ampicillin (50 μg/mL) overnight at 35 °C. It was used as a seed culture (1%), and then the main culture (MagicMedia, Invitrogen, Carlsbad, CA, USA) was grown for 6 h at 30 °C, with orbital shaking at 200 rpm (OSI-500R orbital shaking incubator, TKS, New Taipei City, Taiwan). Subsequently, the target enzyme was produced for 20 h at 25 °C, with orbital shaking at 200 rpm. The cells were harvested by centrifugation (5000× *g* for 15 min at 4 °C) and stored in the freezer at −70 °C.

The recombinant protein was purified with a Profinity eXact purification resin (Bio-Rad) in accordance with the manufacturer’s protocol. The purification was performed in a spin column. After the binding and washing steps, the resin and tagged protein were gently mixed with a potassium fluoride buffer in the spin column on a rocking platform for 30 min at room temperature to perform the on-column cleavage process. Then, the purified tag-free protein was eluted from the spin column. Finally, the purified protein was further concentrated and dialyzed against a 50 mM sodium phosphate buffer (pH 7.0) or 50 mM Tris-HCl buffer (pH 8.0) by 5 times, using a centrifugal filter (Amicon Ultra-15-10 kDa, Millipore, Darmstadt, Germany). Protein concentration was quantified by the Bradford method using bovine serum albumin as the calibration standard [38].

### 2.5. Site-Directed Mutagenesis

To confirm the putative catalytic triad of Est6, site-directed mutagenesis was performed using the overlap extension PCR method. Each amino acid of the catalytic triad was mutated into Ala to evaluate the lipolytic activity. The primers used in site-directed mutagenesis are listed in Table S1. In addition, primers Est6F and Est6R were used for overlap extension PCR in this section. The mutated genes were cloned into the expression vector pPAL7 (Bio-Rad, Hercules, CA, USA). The inserts of recombinant plasmids of site-directed mutants were further confirmed by Sanger sequencing. Then, the confirmed plasmids were transformed into the expression host *E. coli* BL21(DE3) by the heat shock method. The transformants were grown at 35 °C on Spirit Blue Agar plates (50 μg/mL ampicillin, 1% tributyrin, 0.5 µL/mL Tween, and 0.2 mM IPTG) for a rapid enzymatic activity assay. Mutant clones were designated as S135A, D188A, and H219A.

### 2.6. Electrophoresis and Zymographic Analysis

The total cell soluble proteins produced by *E. coli* BL21 Star (DE3)/pPAL7-Est6 and purified Est6 dialyzed and concentrated either in 50 mM sodium phosphate buffer (pH 7.0) or 50 mM Tris-HCl buffer (pH 8.0) were analyzed by electrophoresis using 4–12% sodium dodecyl sulfate-polyacrylamide gel (SDS-PAGE, NuPAGE Novex Bis-Tris precast gel, Invitrogen, Carlsbad, CA, USA) and the MOPS running buffer (NuPAGE MOPS SDS running buffer, Invitrogen, Carlsbad, CA, USA). The protein fractions were visualized with the PageBlue protein staining solution (Fermentas, Vilnius, Lithuania).

Zymographic analysis of the purified Est6 was performed by native PAGE (NativePAGE Novex 3–12% Bis-Tris Gels; NativePAGE Running Buffer, Invitrogen, Carlsbad, CA, USA). After electrophoresis, the gel was laid on an agarose gel prepared with agarose (1.5%) and tributyrin emulsion (1% tributyrin and 0.1% Tween 80) in a 50 mM Tris-HCl buffer (pH 8.0) and was incubated at 4 °C overnight, and the clear bands appeared. The other corresponding gel was visualized with a PageBlue protein staining solution (Fermentas, Vilnius, Lithuania).

### 2.7. Substrate Specificity Measured Using p-Nitrophenyl Esters

Lipolytic activity tests were performed by incubating the enzyme with a 1 mM substrate (stock solution 50 mM in acetonitrile) at 40 °C in 50 mM Tris-HCl buffer (pH 8.0) for 5 min. Eight p-nitrophenyl esters (pNP) with different carbon chain lengths including acetate (C2), propionate (C3), butyrate (C4), valerate (C5), caproate (C6), caprylate (C8), caprate (C10), and laurate (C12) were used to determine the specificity of Est6. For convenience, reaction mixtures (final volume, 150 μL) were prepared in eight strip PCR tubes. The reaction mixtures were transferred immediately to a microplate, and the liberated p-nitrophenol was quantified by measuring the absorbance at 405 nm using a microplate spectrophotometer (PowerWave XS2, Bio-Tek, Winooski, VT, USA). The data were then corrected for nonenzymatic degradation of the ester substrate using an enzyme-free blank of the same reaction mixture treated in the same manner. All samples and blanks were analyzed in triplicate, and activities were quantified by comparison with p-nitrophenol standards in the same buffer. One unit of esterase is defined as the amount of enzyme releasing 1 μmol of free p-nitrophenol per min. The highest enzyme activity on a substrate (pNP acetate) was defined as 100%. Seven different concentrations of pNP acetate (C2) ranging from 0.08 to 1.0 mM were used for the kinetic study, and the K_m_, V_max_, and k_cat_ of Est6 were calculated by the Lineweaver–Burk plot.

### 2.8. Influence of pH and Temperature

The effect of pH on Est6 activity was investigated at 30 °C in various 50 mM buffers (sodium phosphate buffer, pH 5.0–7.0 and Tris-HCl buffer, pH 8.0–10.0). The optimum pH for Est6 activity was measured using pNP acetate as a substrate at 30 °C for 5 min. The absorbance of the reaction mixtures at 348 nm (the pH-independent isosbestic wavelength of p-nitrophenoxide and p-nitrophenol) was measured immediately with a microplate spectrophotometer, as mentioned above. The purified enzyme was maintained at 4 °C for 24 h in various buffers (all with 50 mM) to measure its pH stability. The residual activity was determined under the assay conditions above.

The optimum temperature for Est6 activity was determined analogously by measuring esterase activity at pH 8.0 in the temperature range of 5 °C to 60 °C. After incubation of the purified enzyme in 50 mM Tris-HCl buffer (pH 8.0) for 1 h at varying temperatures ranging from 10 °C to 60 °C, the thermostability of the esterase was measured under standard assay conditions.

### 2.9. Effects of Organic Solvents, Detergents, Metal Ions, Chelators, Inhibitors and NaCl

Est6 was incubated in Tris-HCl buffer (pH 8.0) with 10% (*v/v*) methanol, ethanol, isopropanol, butanol, acetone, acetonitrile, N, N-dimethyl formamide (DMF), or dimethyl sulfoxide (DMSO) at 30 °C for 30 min, and the residual activities were measured. The relative activities were calculated in comparison to the activity of a control without an organic solvent. The enzymes were incubated in 50 mM Tris-HCl buffer (pH 8.0) with different detergents of 1% SDS, Tween 20, Tween 40, Tween 60, Tween 80 or Triton X-100 at 30 °C for 30 min, and then the residual activity was determined under standard conditions. The effects of various chemicals [CaCl_2_, CoCl_2_, CuSO_4_, MgCl_2_, MgSO_4_, MnCl_2_, NiSO_4_, Ethylenediaminetetraacetic acid disodium salt dihydrate (EDTA), phenylmethylsulphonyl fluoride (PMSF)] on the esterase activity were investigated by the addition of the tested compounds at the final concentrations of 1 mM or 10 mM (for EDTA) to the reaction mixture in a 50 mM Tris-HCl buffer (pH 8.0), which was preincubated at 30 °C for 30 min. The remaining activity was then measured according to standard assay conditions. The esterase was incubated in a 50 mM Tris-HCl buffer (pH 8.0) with varying concentrations of NaCl ranging from 0 to 2.5 M at 30 °C for 30 min, and the residual activity of Est6 was measured using pNP acetate as a substrate under standard assay conditions.

### 2.10. Positional Specificity Assay

The positional specificity of Est6 towards triacylglycerol was investigated using a simple continuous spectrophotometric method [39]. A commercially available thioester analog of triacylglycerol, 2,3-dimercapto-1-propanol tributyrate (TBDMP, Sigma-Aldrich, St. Louis, MO, USA), was employed as a substrate. A transparent solution of TBDMP (10 mM) was prepared in a 50 mM Tris-HCl buffer (pH 7.5) with 6% Triton X-100. The absorbance of the 200 μL reaction mixture containing 0.05 mM TBDMP, 0.2 mM 5,5-dithio-bis (2-dinitrobenzoic acid) (DTNB), 1 mM EDTA, 0.045% Triton X-100, a 50 mM Tris–HCl buffer (pH 7.2), and 10 μL of purified Est6 (8 μg) was continuously monitored at 412 nm using a spectrophotometer (Biochrom, Cambridge, United Kingdom) at room temperature for 3 h.

## 3. Results

### 3.1. Molecular Analysis of a New Esterase Gene est6

**Figure 1:**
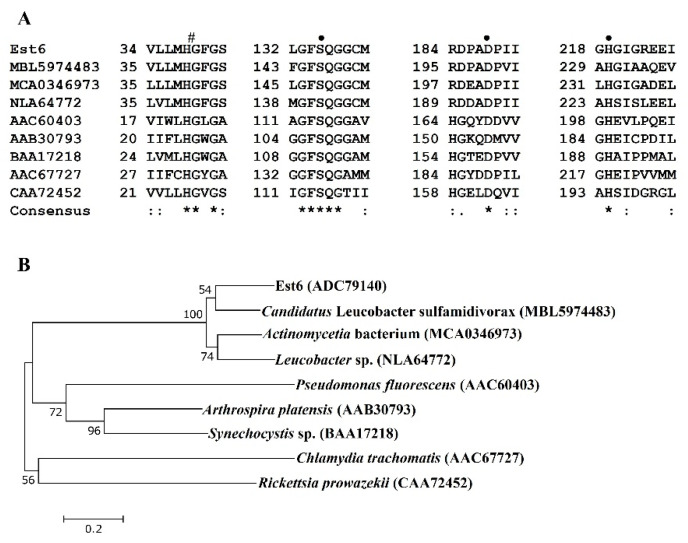
(**A**) Multiple alignments of Est6 and other related proteins: MBL5974483, hypothetical protein D3X82_12175 from *Candidatus* Leucobacter sulfamidivorax; MCA0346973, phospholipase from *Actinobacteria* bacterium; NLA64772, prolyl oligopeptidase family serine peptidase from *Leucobacter* sp.; the following five proteins belonged to family VI bacterial lipolytic enzymes [Arpigny and Jaegar, 1999]: AAC60403, esterase II from *Pseudomonas fluorescens*; AAB30793, serine esterase from *Arthrospira platensis*; BAA17218, serine esterase from *Synechocystis* sp. PCC 6803; AAC67727, predicted lysophospholipase esterase from *Chlamydia trachomatis* D/UW-3/CX; CAA72452, esterase from *Rickettsia prowazekii*. The catalytic triad is indicated as “•” above the sequence, and the oxyanion hole site HG is labeled as “#” above the sequence. All residues are identical (*), there are conserved substitutions (:) and there are semi-conserved substitutions (.) in that column in the alignment. (**B**) Neighbor-joining phylogenetic tree of Est6 and closely related proteins. The analysis was performed using the programs Clustal X 2.0 and MEGA 7.0. The percentage shown from each branch is from the 1000 bootstrap resamplings. Only the bootstrap values higher than 50% were shown. The scale bar represents 0.2 changes per amino acid.

From the ORF analysis of a lipolytic clone ASL06, a new esterase gene *est6* was found. The *est6* gene consisted of 729 bp with G+C content as high as 69.3% and encoded the protein (Est6) of 242 amino acid residues. Based on the BLASTP search, Est6 shared 65.48% identity with the highest score to the hypothetical protein (MBL5974483.1) from *Candidatus* Leucobacter sulfamidivorax. The other closely related proteins to Est6 were predicted from the whole genome sequencing data of several microbes or metagenomes of environmental samples. These microbes and their related proteins, with accession numbers shown in parentheses, included *Actinomycetia* bacterium (MCA0346973), *Leucobacter* sp. (NLA64772.1), *Microbacteriaceae* bacterium (NLB47291), *Leucobacter soli* (WP_218114315), *Leucobacter weissii* (WP_208098032), and *Klugiella xanthotipulae* (WP_246054504), which affiliated with the bacterial phylum of *Actinobacteria* with high G+C content. Est6 also harbored the GFSQG motif (Figure 1A), which is one of the characteristics of the family VI bacterial lipolytic enzymes. According to the multiple sequence alignment of Est6 and its closely related proteins, the predicted oxyanion hole site (His39Gly40) and catalytic triad (Ser135, Asp188 and His219) could be obtained [3,40]. From the phylogenetic tree (Figure 1B), Est6 and three closely related proteins can be classified into a new group of family VI bacterial lipolytic enzymes, instead of the group of five proteins suggested by Arpigny and Jaeger (1999) [3]. In addition, Est6 was recognized as a non-secretory protein and calculated to have a pI of 4.80 and a molecular mass of 26.1 kDa, which also correlated well to the range 23–26 kDa of family VI lipolytic enzymes [3]. Moreover, Est6 had a predicted scaled solubility value of 0.614, which is greater than 0.45, so Est6 was predicted to be a soluble protein based on the SOLpro analysis [31].

### 3.2. Overexpression, Purification and Electrophoretic Analysis of Est6

**Figure 2:**
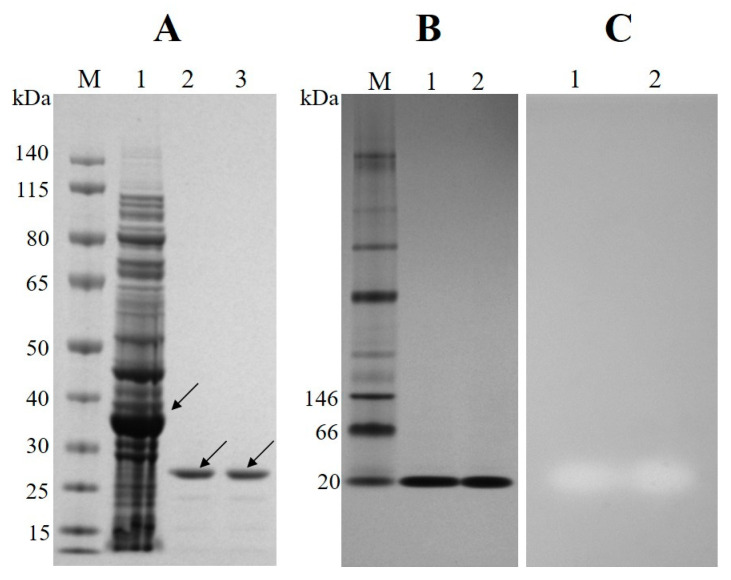
(**A**) SDS-PAGE analysis of overexpressed and Profinity eXact purified Est6 in *E. coli* BL21 Star (DE3). Samples were run on a 4–12% NuPAGE Novex Bis-Tris mini gel. Lane M, protein molecular mass ladder (Fermentas); lane 1, total soluble cell protein; lane 2, Est6 in 50 mM sodium phosphate buffer (pH 7.0); lane 3, Est6 in 50 mM Tris-HCl buffer (pH 8.0). The arrows indicate the Profinity eXact tagged Est6 and tag-free Est6, respectively. (**B**) Native PAGE analysis of purified Est6. Samples were run on a NativePAGE Novex 3–12% Bis-Tris gel. Lane M, native protein mass ladder (NativeMark unstained protein standard, Invitrogen). Lane 1, Est6 in 50 mM sodium phosphate buffer (pH 7.0); lane 2, Est6 in 50 mM Tris-HCl buffer (pH 8.0). (**C**) Zymogram of purified Est6 on an agarose gel containing 1% tributyrin. The sample order is as same as that in (**B**).

Est6 was produced as an N-terminal Profinity eXact-tag fusion protein in a soluble form using a pPAL7 expression vector in *E. coli* BL21 Star (DE3) cells. After purification with single-step chromatography using the Profinity eXact resin, the tag of the fusion protein was removed. SDS-PAGE analysis indicated that the protein was homogeneous and tag-free and that the molecular mass of Est6 was approximately matched to the predicted size (Figure 2A). Moreover, after native PAGE analysis, the purified Est6 showed a single band on the native gel when Est6 was incubated in the Tris-HCl buffer (pH 8.0) (Figure 2B). Furthermore, a clear band appeared around the same places corresponding to native PAGE when the zymographic analysis was performed as described in the Methods (Figure 2C). Based on the SDS-PAGE and native PAGE analyses, Est6 was recognized to exist in a monomeric form according to their estimated molecular mass.

### 3.3. Activity of Est6 on Short-Chain Fatty Acid Substrates

Various p-nitrophenyl acyl esters with different chain lengths (acetate, C2; propionate, C3; butyrate, C4; valerate, C5; caporate, C6; caprylate, C8; caprate, C10, laurate, C12) were used to examine substrate specificity. The substrate specificity of Est6 decreased with an increase in the acyl chain length up to C12 (Table 1). Among the eight pNP esters tested, Est6 showed its highest activity on the substrate pNP acetate (Table 1). Based on the results above, Est6 could be recognized as an esterase but not a lipase. The Lineweaver–Burk plot was drawn according to the result of the kinetic study with pNP acetate (C2) as a substrate (Figure S1). The K_m_, k_cat_, and k_cat_/K_m_ values were 1.07 mM, 10.46 s^−1^, and 9.79 mM^−1^ s^−1^, respectively.

### 3.4. Influence of pH and Temperature on Est6 Activity and Stability

**Figure 3:**
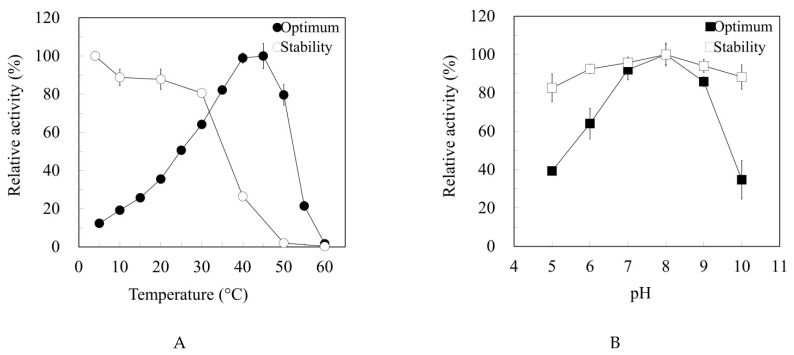
Effect of pH and temperature on activity and stability of Est6. (**A**) The enzyme activity was measured at an absorbance of 348 nm in various pH buffers at 30 °C. The value obtained at pH 8.0 was taken as 100%. Optimal pH tests were displayed as filled squares. The pH stability of the enzyme preserved in various pH buffers for 24 h at 4 °C was demonstrated as open square. The enzyme activity was measured at an absorbance of 348 nm in a pH 8.0 buffer at 30 °C. Error bars indicate standard deviations. (**B**) The enzyme activity was measured at various temperatures at pH 8.0 under standard assay conditions. The relative activity obtained at 45 °C was taken as 100%. The test for the optimal temperature of Est6 was shown as a filled circle, while the thermostability of the enzyme at different temperatures was displayed as an open circle. The residual activity was determined after incubation of the enzyme at different temperatures for 1 h. The activity measurement was carried out using the standard enzyme assay. All samples and blanks were analyzed in triplicate. The relative activity of the enzyme preserved at 4 °C was taken as 100%. Error bars indicate standard deviations.

The purified Est6 was active in the broad range of pH 5.0 to pH 10.0, with maximal activity at pH 8.0 (Figure 3A). The enzyme maintained more than 80% relative activity in the pH range of 5.0 to 10.0 after preincubation at 4 °C for 24 h (Figure 3A). Est6 displayed the highest activity at 45 °C, with activity rapidly decreasing at temperatures above 55 °C. Between the temperatures of 25 °C to 50 °C, more than 50% of the maximal activity that was observed at 45 °C remained (Figure 3B). Thermostability analysis by preincubation at various temperatures for 1 h revealed that Est6 was unstable at 40 °C and rapidly inactivated at 60 °C (Figure 3B).

### 3.5. Effect of Organic Solvents, Metal Ions, Detergents, PMSF, and NaCl on the Activity of Est6

**Figure 4:**
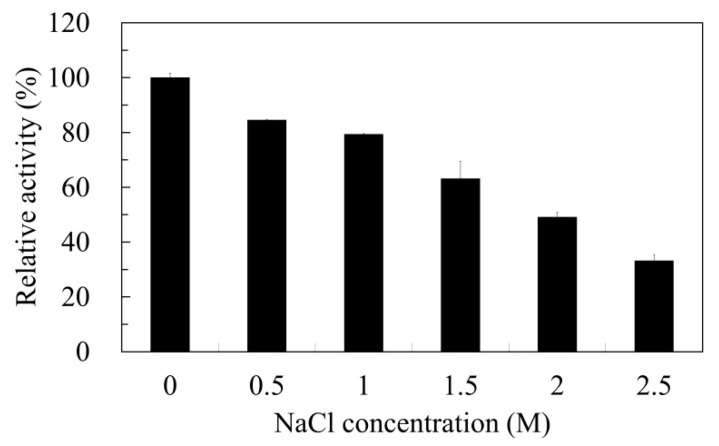
Effect of NaCl concentrations on Est6 activity. The residual activity was determined after incubation of the enzyme in the Tris-HCl buffer (pH 8.0) with different concentrations of NaCl at 30 °C for 30 min. The activity measurement was carried out using the standard enzyme assay. All samples and blanks were analyzed in triplicate. The relative activity of Est6 in the reaction buffer without NaCl was defined as 100%. Error bars indicate standard deviations.

The stability of Est6 in various organic solvents was determined (Table 2). Est6 activity was stable in 10% methanol, 10% ethanol, 10% isopropanol, or 10% DMSO, with approximately 70% residual activity compared with that of a non-organic solvent condition. Est6 showed very low activity (0.3%) when incubated in 10% Butanol. The activity of Est6 was severely inhibited by Cu^2+^, Ni^2+^, or Co^2+^ ions; however, the activity maintained the same levels in the presence of Mg^2+^, Ca^2+^, or Mn^2+^, while EDTA slightly activated the enzyme (Table 3). The effect of PMSF, a serine modifier, on the activity of Est6 was determined. The result indicated that at 1 mM PMSF, the catalytic serine residue of Est6 was modified and its activity was lost (Table 3). In the presence of 1% SDS, the remaining activity of Est6 was barely detected, while the enzyme maintained more than 60% of its relative activity in the presence of 1% Tween 20, Tween 40, Tween 60, Tween 80, and Triton X-100 at 30 °C for 30 min, respectively, compared with that of a non-detergent condition (Table 3). In addition, the stability of Est6 activity was examined after incubation in various concentrations of NaCl at 30 °C for 30 min. The relative activity of Est6 decreased with increasing NaCl concentration and remained at approximately 50% at the 2 M NaCl concentration compared with that of a no-NaCl condition (Figure 4).

### 3.6. Positional Specificity of Est6

Both stereospecificity and regiospecificity are important characteristics of lipases/esterases when used for synthesizing fine chemicals [4]. The positional specificity of Est6 towards triacylglycerol was analyzed using a simple continuous spectrophotometric method. The OD412 nm value for TBDMP hydrolysis ranged from 0.6 to 0.7, approximately half the theoretical value (1.250) [37] for the complete hydrolysis of TBDMP, indicating that Est6 hydrolyzed only one of the TBDMP thioester groups. The result inferred that Est6 had the positional specificity towards triacylglycerol.

### 3.7. Site-Directed Mutagenesis of the Catalytic Triad of Est6

**Figure 5:**
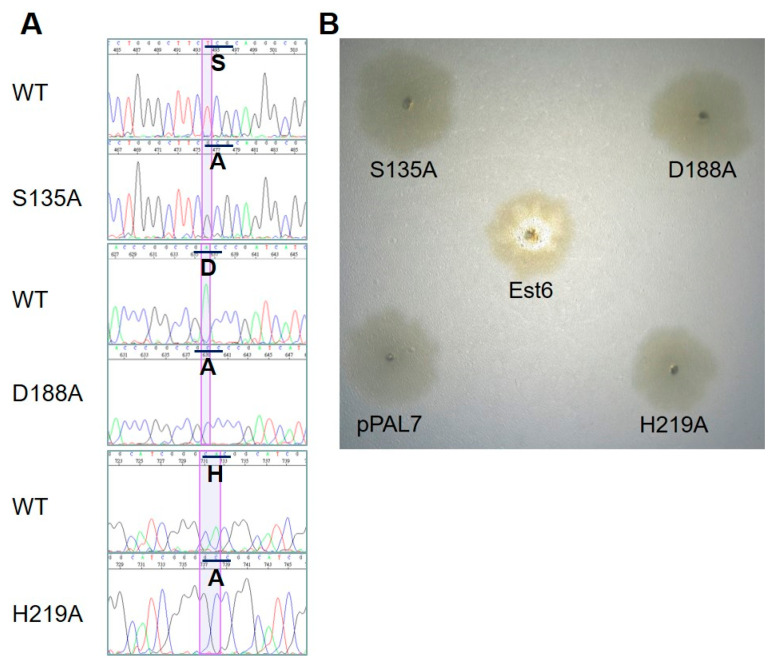
Site-directed mutagenesis of the predicted catalytic triad of Est6. (**A**) DNA sequencing of the predicted catalytic triad regions of wild type and related mutants. The codons and amino acids are shown in underscore and bold form, respectively. (**B**) Lipolytic plate assay for the wild type and related mutants. Mutant colonies show no clear halos compared with the wild type Est6. The transformant harboring the expression vector pPAL7 is the negative control.

Each residue of the putative catalytic triad of Est6 was mutated to Ala successfully by an overlap extension PCR and confirmed by DNA sequencing (Figure 5A). Evaluating the lipolytic activity of each mutant was monitored on the Spirit Blue Agar plate. After two days of incubation at 35 °C and one day in the refrigerator, all mutants had no lipolytic activity by checking clear halos around or in the colonies compared with wild-type Est6 (Figure 5B).

### 3.8. Structural Modeling of Est6

**Figure 6:**
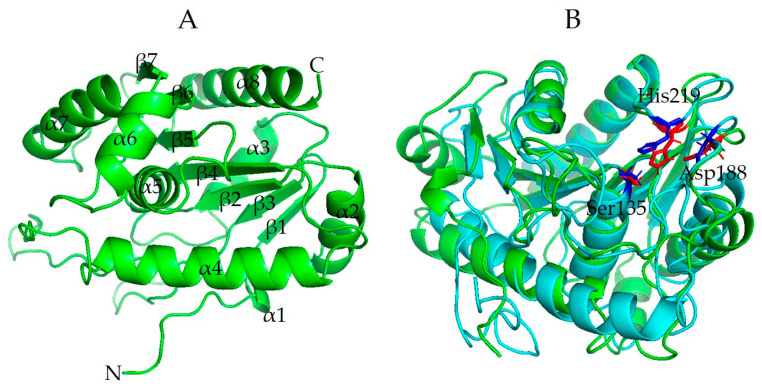
3D structural modeling of Est6. (**A**) The rank 1 model of Est6 with eight α-helices and seven β-sheets predicted by AlphaFold2. (**B**) Superposition of the rank 1 model predicted by AlphaFold2 (in green) with the rank 1 model predicted by I-TASSER (in cyan). The putative catalytic triad, including Ser135, Asp188, and His219, are shown in red stick forms predicted by AlphaFold2 and in blue stick forms predicted by I-TASSER, respectively. PyMOL2 software was used to visualize and superpose the predicted 3D structural models.

The secondary structure of Est6 was predicted to have 10 α-helices and eight β-sheets by PSIPRED 4.0 (Figure S2). However, Est6 was estimated to possess six α-helices and six β-sheets based on the top 10 threading templates by I-TASSER (Figure S3). For 3D structural analysis, the top five final models of Est6 predicted by I-TASSER were also obtained. The C-score and estimated TM-score for model 1 were -0.66 and 0.63, respectively. Est6 was predicted to have six α-helices and seven β-sheets based on a 3D model (Figure S4A). The best threading template for the three-dimensional modeling of Est6 was a carboxyl esterase from *Rhodobacter sphaeroides* [PDB code: 4fhzA] with the highest TM-score of 0.853 and the lowest root-mean-square deviation (RMSD) value of 1.44 used by I-TASSER (Figure 4B). The residues of the putative catalytic triad, consisting of Ser135, Asp188, and His219, are located in the coils between the α/β domains (Figure S4C). Especially for the nucleophilic attack of the active site Ser135, it is located in the close junction of the β3 and α3 domains. Asp188 and His219 are located on the loops between β5-α5 and β6-α6, respectively. Furthermore, AlphaFold2 is a state-of-the-art tool for protein structural modeling. The top five models obtained by AlphaFold2 analyses show pLDDT (predicted local distance difference test) and predicted TM-score values ranging from 95.2 to 95.9 and 0.928 to 0.936, respectively. The rank 1 model for Est6 was predicted to comprise eight α-helices and seven β-sheets, which is different from the prediction by I-TASSER (Figure 6A). Both the rank 1 models of Est6 predicted by AlphaFold2 and I-TASSER were superposed by PyMOL2, showing the lowest root-mean-square deviation (RMSD) value of 2.205 (Figure 6B). The residues of the putative catalytic triad were also predicted to be in similar regions between the α and β domains (Figure 6B). Based on the predictions by I-TASSER and AlphaFold2, no lid domain was found to cover the catalytic triad of Est6.

## 4. Discussion

In the present study, a new esterase gene *est6* was discovered from an activated sludge metagenome of a swine wastewater treatment facility, and its gene product Est6 was characterized. The *est6* gene with G+C content as high as ca. 70% could be overexpressed to produce a soluble form of Est6 in *E. coli*. The primary sequence analysis of Est6 showed the presence of pentapeptide GFSQG, which corresponded with the conserved GXSXG motif in the α/β hydrolase superfamily that includes many lipolytic enzymes, proteases, dehalogenases, peroxidases, and epoxide hydrolases [41]. Phylogenetic analysis also revealed that Est6 belongs to family VI esterases [3] and forms a new subfamily together with its closely related proteins group (Figure 1B). Since most lipolytic enzymes of family VI are not well characterized, very little is known about this family. To the authors’ best knowledge, this is the first report of a new member of typical family VI lipolytic enzymes screened from a metagenomic library, produced, purified, and biochemically characterized.

As indicated by the data for the optimum enzymatic activity, Est6 was recognized as an alkaline esterase, which also showed salt tolerance to 2 M NaCl with 50% activity retained. Previous studies indicated that the rich negatively charged amino acid residues on the surface of these proteins may contribute to salt tolerance [42,43,44]. Est6 contains 13.6% (by frequency) of negatively charged amino acids from primary sequence analysis, and the conserved negatively charged residues are located on the surface of the protein by performing the modeling of the 3D structure with I-TASSER server (data not shown) [35]. On the other hand, Est6 is inferior to those esterases: EstKT4, EstKT7, EstKT9 [45], and EstWSD [46] in salt tolerance. The temperature and pH for the maximal activity of Est6 were 45 °C and 8.0, respectively. In the original sample source of activated sludge from a swine wastewater treatment facility, the temperature and pH were 30 °C and 7.6, respectively, which are good environmental factors for Est6 activity. Est6 maintained only 9.3% activity in the presence of 1 mM PMSF, and this result suggests that Est6 is a serine esterase with a Ser135 active site and without a lid domain. The finding is consistent with the 3D modeling. The chelator EDTA had no influence on Est6 activity, indicating that Est6 doesn’t require metal ions as the cofactor for enzyme activity. This finding is similar to many studies [9,11,16,47]. Both stereospecificity and regiospecificity are important characteristics of lipases/esterases when used for synthesizing fine chemicals [4]. A novel lipase derived from a Korean tidal flat metagenomic library was determined to be non-regiospecific towards triacylglycerol in the spectrophotometric analysis [48]. Another esterase ES46.5K derived from mouse hepatic microsomes showed stereospecific and regioselective hydrolysis of cannabinoid esters [49]. In general, microbial lipases with 1,3-specific activity towards triacylglycerol are more familiar to be recovered [50]. In the present study, Est6 affiliated with family VI as one of the carboxylesterases was regiospecific to hydrolyze triacylglycerol.

Esterase II from *P. fluorescens* and EstRag from *Lysinibacillus* sp. were characterized as members of the family VI lipolytic enzymes [6,11]. Est6 is similar to esterase II and EstRag which showed a preference for short chain lengths of pNP esters. However, the relative activities of three esterases to short chain lengths of pNP esters were different. The activity of each esterase was maintained or increased when incubated with EDTA and Ca^2+^. Esterase II and Est6 were inhibited by PMSF in activity. Another characterized family VI esterase, EstA6, from *Pseudomonas* sp. CR-611 differed in many aspects of biochemical properties from Est6 [9]. However, the active forms of EstA6 and esterase II are dimeric when they are purified from the parental microbial strains. It is suggested that the appropriate formation of such dimeric forms would be enhanced under some internal environment in *Pseudomonas* sp., acting as a shield for the hydrophobic substrate-binding site [9]. Nevertheless, the purified Est6 from recombinant clones was a monomer, such as that of EstA6. Since Est6 was derived from a metagenome and shared moderate identity with the closest proteins in the non-redundant protein sequence database of the NCBI, the parental microbial strain of Est6 has not been found through BLAST search up to now. Therefore, the active form of Est6 produced by its parental microbial strain is unknown.

Another three novel esterases from activated sludge metagenomes have been discovered and characterized biochemically [20,21,23]. Lipo1 (ABR68854) affiliated with family IV lipolytic enzymes is low-temperature-adapted and exhibits its highest activity at a temperature of 10 °C and pH 7.5, while EstAS (ACJ13070) belongs to family III lipolytic enzymes and displays maximum activity at a temperature and pH of 35 °C and 9.0, respectively. Est-XG2 (AGS38342), a thermophilic esterase, belonging to family VII exhibits optimum activity at pH 8.5 and 70 °C and moderate tolerance to organic solvents and surfactants. Moreover, three esterases are expected to be useful for biotechnological applications because of their unique biochemical properties. The possible reason is that microorganisms inhabiting activated sludge are employed to decompose organic matter in wastewater. Therefore, it is very likely to isolate new esterases with excellent properties for biotechnological applications.

In summary, the activated sludge metagenome is a valuable reservoir for screening novel esterases. The *est6* gene was isolated from an activated sludge metagenome of a swine wastewater treatment facility and its gene product Est6 possesses noteworthy biochemical characteristics, showing activity over a wide range of temperatures and pH as well as in various organic solvents, metal divalent cations, and detergents. Therefore, these characteristics might extend the potential biotechnological applications of Est6.

## Figures and Tables

**Table 1:** Substrate specificity of the purified Est6.

Substrate pNP ester	Specific Activity (U/mg)	Relative Activity (%)
Acetate (C2)	17.7 ± 1.3	100.0 ± 7.3
Propionate (C3)	11.8 ± 0.9	66.7 ± 5.1
Butyrate (C4)	10.2 ± 0.5	57.6 ± 2.8
Valerate (C5)	9.5 ± 0.5	53.7 ± 2.8
Caproate (C6)	6.0 ± 0.3	33.9 ± 1.7
Caprylate (C8)	1.5 ± 0.1	8.5 ± 0.6
Caprate (C10)	0.4 ± 0.0	2.3 ± 0.0
Laurate (C12)	0.1 ± 0.1	0.6 ± 0.6

Esterase activity was measured at 40 °C for 5 min in the presence of the 50 mM Tris-HCl buffer (pH 8.0). All samples and blanks were measured in triplicate. Values are shown in mean ± SD. The relative activity of Est6 in the reaction buffer with pNP acetate as a substrate was defined as 100%.

**Table 2:** Effects of organic solvents on the stability of Est6.

Solvent (10%)	Relative Activity (%)
Methanol	97.0 ± 3.0
Ethanol	85.5 ± 3.4
Isopropanol	83.8 ± 3.8
Butanol	0.3 ± 0.0
Acetone	43.0 ± 2.7
Acetonitrile	29.1 ± 0.5
DMF	38.9 ± 2.5
DMSO	69.9 ± 5.8

Est6 was incubated in Tris-HCl buffer (pH 8.0) with 10% (*v/v*) organic solvent at 30 °C for 30 min, and the residual activities were measured at 30 °C for 5 min in presence of a 50 mM Tris-HCl buffer (pH 8.0). All samples and blanks were analyzed in triplicate. Values are shown in mean ± SD. The relative activity of Est6 in the reaction buffer without organic solvent was defined as 100%.

**Table 3:** Effects of various compounds on the stability of Est6.

Compound	Relative Activity (%)
Cation (1 mM)	
CaCl_2_	99.5 ± 1.8
MgCl_2_	95.9 ± 5.6
MgSO_4_	99.9 ± 0.6
MnCl_2_	95.5 ± 4.3
CoCl_2_	12.4 ± 0.9
NiSO_4_	8.6 ± 0.3
CuSO_4_	1.2 ± 0.1
Chelator	
EDTA (1 mM)	109.6 ± 1.9
EDTA (10 mM)	100.9 ± 2.4
Inhibitor (1 mM)	
PMSF	9.3 ± 0.1
Detergent (1%)	
SDS	0.5 ± 0.5
Tween 20	88.1 ± 2.3
Tween 40	71.0 ± 5.0
Tween 60	68.0 ± 1.6
Tween 80	81.1 ± 5.3
Triton X-100	60.0 ± 3.2

Est6 was incubated in a Tris-HCl buffer (pH 8.0) with additives at 30 °C for 30 min, and the residual activities were measured at 30 °C for 5 min in the presence of a 50 mM Tris-HCl buffer (pH 8.0). All samples and blanks were analyzed in triplicate. Values are shown in mean ± SD. The relative activity of Est6 in the reaction buffer without any additive was defined as 100%.

## Data Availability

Not applicable.

## Supplementary Materials

The following supporting information can be downloaded at: https://www.mdpi.com/article/10.3390/microorganisms10122403/s1, Table S1: The primers used in site-directed mutagenesis of catalytic triad in this study; Figure S1: Lineweaver–Burk plot of Est6 with pNP acetate (C2) as a substrate; Figure S2: The secondary structure of Est6 predicted by PSIPRED 4.0; Figure S3: The secondary structure of Est6 predicted by I-TASSER; Figure S4: 3D structural modeling of Est6 by I-TASSER.
