# Supplementary material for: Molecular Cloning and Characterization of a New Family VI Esterase from an Activated Sludge Metagenome
Source: Microorganisms. 2022 Dec 4;10(12):2403. doi: 10.3390/microorganisms10122403 (PMC9786865; doi:10.3390/microorganisms10122403)
Supplement: Supplementary file 1 [file microorganisms-10-02403-s001.zip › microorganisms-2053893-supplementary.pdf]

Table S1. The primers used in site-directed mutagenesis of catalytic triad in this study.

Primer	Sequence (5′ to 3′)	Description
S135AF	GTGCCCCTGGGCTTCgCGCAGGGCGGCTGCATG	Each pair of forward and reverse primers are complementary, modified codons in the forward primers are blocked in yellow, and mutated nucleotides are in lowercase.
S135AR	CATGCAGCCGCCCTGCGcGAAGCCCAGGGGCAC	
D188AF	GGGCGCGACCCGGCCGcCCCGATCATCGAAGCAG	
D188AR	CTGCTTCGATGATCGGGgCGGCCGGGTCGCGCCC	
H219AF	CTACCACGGCATCGGGgcCGGCATCGGGCGCGAG	
H219AR	CTCGCGCCCGATGCCGgcCCCGATGCCGTGGTAG	

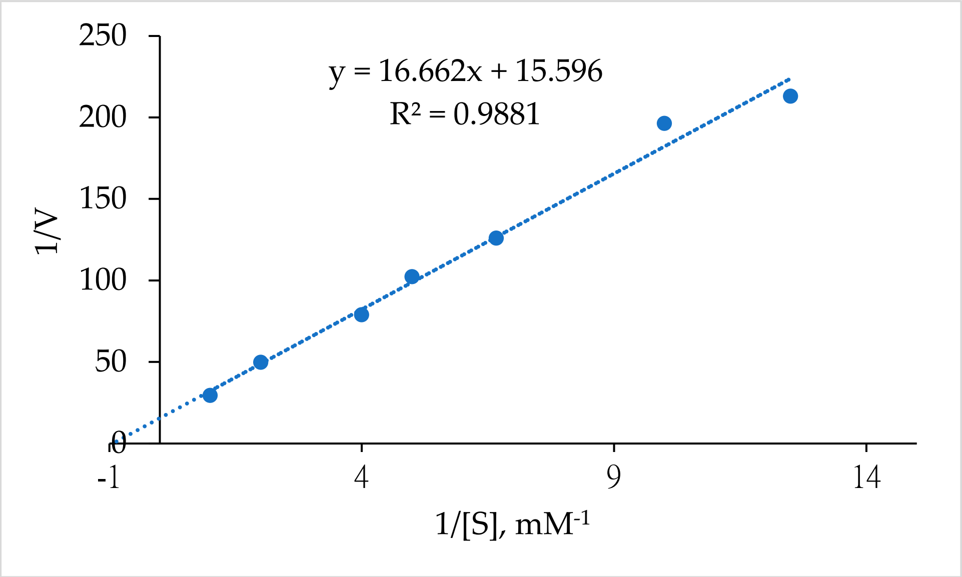

Figure S1. Lineweaver–Burk plot of Est6 with pNP acetate (C2) as a substrate. Seven different substrate concentrations ranging from 0.08, 0.1, 0.15, 0.20, 0.25, 0.5, and 1.0 mM were used for the kinetic study.

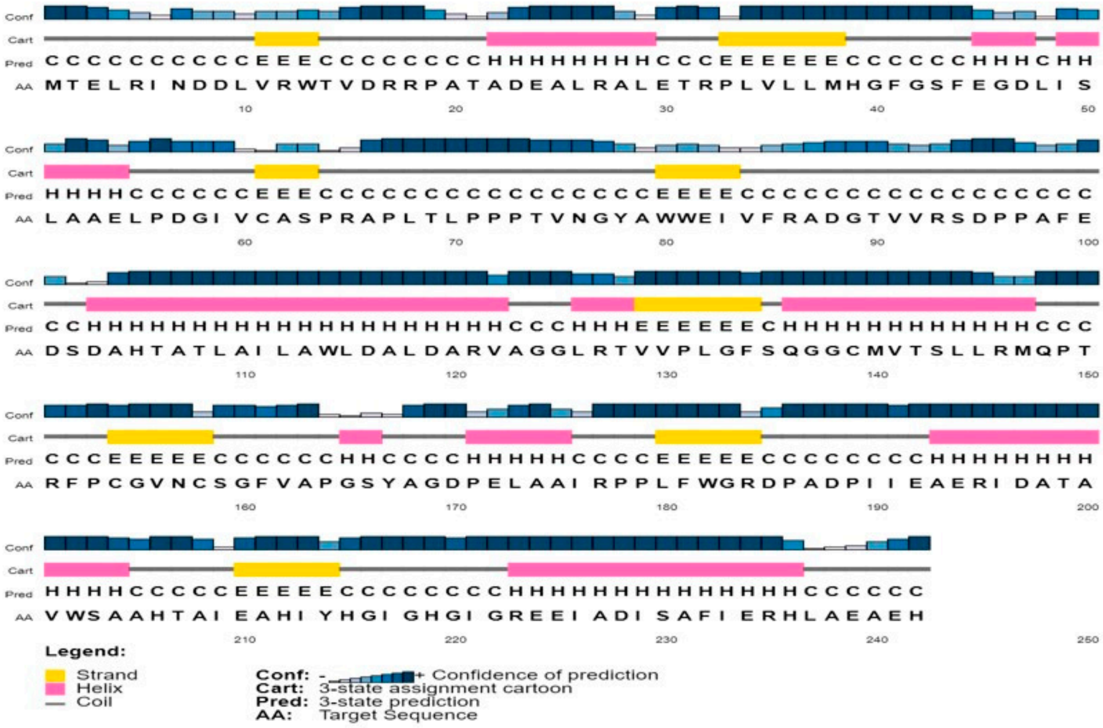

Figure S2. The secondary structure of Est6 predicted by PSIPRED 4.0. Est6 was estimated to have ten α-helices and eight β-sheets.

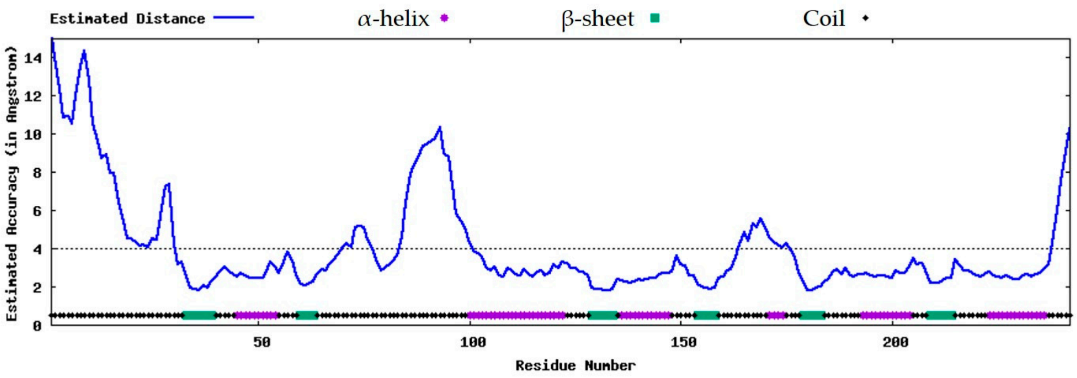

Figure S3. The secondary structure of Est6 predicted by I-TASSER. Est6 was estimated to have six α-helices and six β-sheets.

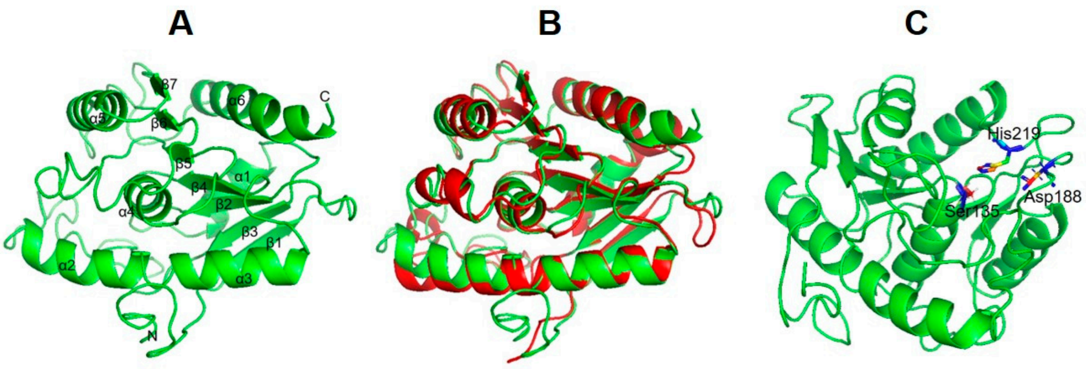

Figure S4. 3D structural modeling of Est6 by I-TASSER. (A) The top one model of Est6 with six α-helices and seven β-sheets. (B) Superposition of Est6 model (in green) with the carboxyl esterase [PDB code: 4fhzA, in red] of *Cereibacter sphaeroides* as the protein structure template with an RMSD value of 1.44. (C) The putative catalytic triad including Ser135, Asp188, and His219 shown in ball-and stick forms, respectively. PyMOL2 software was used to visualize the predicted 3D structural models.
